# Impact of Multidomain Lifestyle Intervention on Cerebral Cortical Thickness and Serum Brain-Derived Neurotrophic Factor: the SUPERBRAIN Exploratory Sub-study

**DOI:** 10.1007/s13311-022-01276-x

**Published:** 2022-08-01

**Authors:** So Young Moon, Sohui Kim, Seong Hye Choi, Chang Hyung Hong, Yoo Kyoung Park, Hae Ri Na, Hong-Sun Song, Hee Kyung Park, Muncheong Choi, Sun Min Lee, Buong-O Chun, Jong-Min Lee, Jee Hyang Jeong

**Affiliations:** 1 Department of Neurology, Ajou University School of Medicine, Suwon, Korea; 2 Department of Electronic Engineering, Hanyang University, Seoul, South Korea; 3 Department of Neurology, Inha University School of Medicine, Incheon, Korea; 4 Department of Psychiatry, Ajou University School of Medicine, Suwon, Korea; 5 Department of Medical Nutrition, Graduate School of East-West Medical Nutrition, Kyung Hee University, Suwon, Korea; 6 Department of Neurology, Bobath Memorial Hospital, Seongnam, Korea; 7 Department of Sports Sciences, Korea Institute of Sports Science, Seoul, Korea; 8 Department of Neurology, Ewha Womans University School of Medicine, 260 Gonghang-daero, Gangseo-gu, Seoul, 07804 Korea; 9 Department of Physical Education, Kookmin University, Seoul, Korea; 10 Exercowork, Seoul, Korea; 11 Department of Biomedical Engineering, Hanyang University, Sanhak-kisulkwan Bldg, #319, 222 Wangsipri-ro, Sungdong-gu, Seoul, 133-791 Republic of Korea

**Keywords:** Cortical thickness, Dementia, Prevention, Lifestyle, Intervention

## Abstract

In the SoUth Korean study to PrEvent cognitive impaiRment and protect BRAIN health through lifestyle intervention in at-risk elderly people (SUPERBRAIN), we evaluated the impact of a 24-week facility-based multidomain intervention (FMI) and home-based MI (HMI) on cortical thickness, brain volume, and the serum brain-derived neurotrophic factor (BDNF). Totally, 152 participants, aged 60–79 years without dementia but with ≥ 1 modifiable dementia risk factor, were randomly assigned to the FMI, HMI, or control groups. Among them, 55 participants (20 FMI, 19 HMI, and 16 controls) underwent brain MRI at baseline and 24 weeks. We compared changes in global/regional mean cortical thickness at the region-of-interest (ROI) between the intervention and control groups. The changes in the total cortical gray matter volume and global mean cortical thickness were compared using analysis of covariance with age, sex, and education as covariates. ComBat site harmonization was applied for cortical thickness values across the scanners. ROI-based analysis was controlled for multiple comparisons, with a false discovery rate threshold of *p* < 0.05. Serum BDNF levels were significantly higher in the FMI group than in the control group (*p* = 0.029). Compared with the control group, the mean global cortical thickness increased in the FMI group (0.033 ± 0.070 vs. − 0.003 ± 0.040, *p* = 0.013); particularly, cortical thickness of the bilateral frontotemporal lobes, cingulate gyri, and insula increased. The increase in cortical thickness and serum BDNF in the FMI group suggests that group preventive strategies at the facility may be beneficial through structural neuroplastic changes in brain areas, which facilitates learning and neurotrophic factors.

**Supplementary Information:**

The online version contains supplementary material available at 10.1007/s13311-022-01276-x.

## Introduction

Recent studies have shown that lifestyle modifications can prevent the occurrence of dementia in at-risk individuals [1–3]. The Finnish Geriatric Intervention Study to Prevent Cognitive Impairment and Disability (FINGER), which included dietary counseling, physical exercise, cognitive training, and vascular and metabolic risk monitoring, was the first large randomized controlled trial to report beneficial effects on cognition for a 2-year multidomain lifestyle intervention among older individuals with an increased risk of dementia [4]. Although the French Multidomain Alzheimer Preventive Trial (MAPT) [5] and the Dutch Prevention of Dementia by Intensive Vascular Care (PreDIVA) trial [6] reported a lack of effect on the primary outcomes, exploratory subgroup analyses in both studies have provided evidence that interventions yielded cognitive benefits in subpopulations of participants with increased risk of dementia [6, 7]. Furthermore, the SoUth Korean study to PrEvent cognitive impaiRment and protect BRAIN health through lifestyle intervention in at-risk elderly people (SUPERBRAIN) proved for the first time that the multidomain intervention based on the FINGER study, modified according to the culture and circumstances of the other country, is feasible and may be effective [8–10].

In contrast to the cognitive benefits, the effects of multidomain lifestyle interventions on neural substrates underling the cognitive benefits are still not fully clear. The possible mechanisms of dementia prevention in at-risk individuals by multidomain lifestyle intervention involve enhancing or maintaining cognitive reserve as well as risk reduction of potentially modifiable risk factors [1]. Cognitive reserve is related to the efficiency of the brain functions and its neural substrate is referred to brain volume, the intensity of cerebral metabolism, or the connectivity of neural networks such as density of synapses and dendrites branching [11]. Therefore, enhanced or maintained cognitive reserve by multidomain lifestyle interventions could be evaluated by changes in structural or functional brain imagings or concentration of neurotrophic factors such as the brain-derived neurotrophic factor (BDNF). As such, physical activity has been associated with slower rates of cognitive decline and lower risk of dementia [12], and with increased levels of peripheral serum or plasma BDNF [13], and social interactions [14] and environmental enrichment [15] improve memory deficits in AD-like animal models through BDNF-dependent hippocampal neurogenesis, the exploratory analysis of the SUPERBRAIN [10] also showed that serum BDNF levels were significantly increased in the facility-based multidomain intervention (FMI) compared to the control group. However, benefits on cortical thickness or brain volumes of multidomain lifestyle interventions remain unclear.

A few lifestyle-based trials have so far included brain MRI markers for cortical thickness, or brain volumes. While randomized controlled trials assessing 6- to 24-month single-domain lifestyle interventions including physical activity [16, 17], a multimodal social engagement program [18], or nutrition-related interventions [19, 20] have reported promising effects on various gray matter measures including Alzheimer’s disease (AD) signature regions such as entorhinal, inferior temporal, middle temporal, and fusiform regions on MRI. However, the FINGER MRI exploratory sub-study did not show significant differences between the intervention and control groups in terms of changes in brain volumes or regional cortical thickness including AD signature regions after 2 years in at-risk elderly individuals without substantial impairment [21].

In this study, we aimed to evaluate the impact of a 6-month multidomain lifestyle intervention on the neural substrates related to the cognitive reserve through exploratory analyses of changes in global and regional cortical thickness, brain volumes, and the serum BDNF in the SUPERBRAIN.

## Methods

### Study Population

The SUPERBRAIN trial protocol (ClinicalTrials. gov: NCT03980392) [8] and primary findings [10] have been previously described in detail. In brief, this study was a 24-week, multicenter, outcome assessor-blinded, randomized controlled trial with a three-parallel-arm design performed in three hospitals and five public health centers across South Korea. The facility-based multidomain intervention (FMI) and home-based multidomain intervention (HMI) groups were the two experimental arms, and the control group was used as a comparator. The participants were 152 individuals aged 60–79 years who had no dementia and one or more modifiable dementia risk factors such as hypertension [22], diabetes mellitus [23], dyslipidemia [24], smoking [25], obesity [26], abdominal obesity [27], metabolic syndrome [28], educational level of ≤ 9 years, social isolation [8], and physical inactivity [8]. In addition, they had a Mini-Mental State Examination [29] *z* score of ≥  − 1.5, were able to perform independent activities of daily living and pass a literacy test [30], and had a reliable informant who can provide investigators with the requested information. Individuals with dementia, conditions affecting safe participation or cooperation, or concurrent participation in another trial were excluded. This study was conducted in accordance with the International Conference on Harmonization Good Clinical Practice Guidelines. The institutional review boards of all institutions approved the protocol and consent forms before the start of the study. Written informed consent was obtained from all potential participants by a doctor before enrollment in the study.

**Fig. 1:**
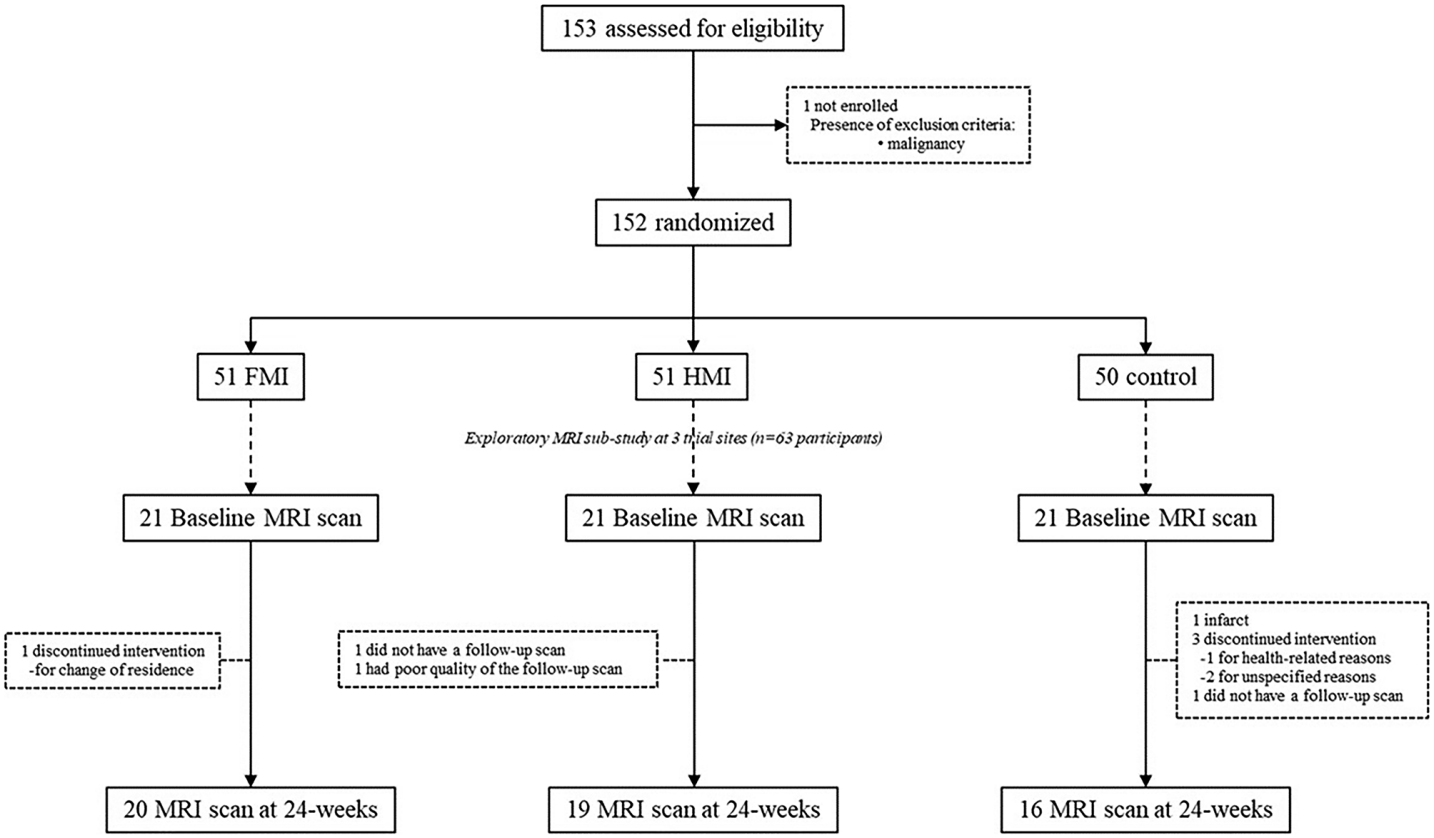
Diagram for exploratory MRI analyses in the SUPERBRAIN trial. FMI facility-based multidomain intervention, HMI home-based multidomain intervention

The SUPERBRAIN MRI exploratory sub-study included 63 participants from three trial sites (Ajou University Hospital, Ewha Woman’s University Medical Center, and Inha University Hospital), where MRI resources were available (Fig. 1). Brain scans were conducted at the baseline and 24-week visits. The present study included 55 participants with both baseline and repeat scans of good quality for brain cortical thickness analysis, which was evaluated by a subjective step and one participant’s follow-up scan was excluded because the gray matter surfaces were shown to be overlapped by the skull during the MRI processing for the MRI structural analysis.

### Randomization and Intervention

The participants were randomly assigned to the FMI, HMI, and control groups at baseline in a 1:1:1 ratio. Randomization was performed using a permuted block randomization method with block sizes of three and six using SAS macro programming and stratified by the participating center. Therefore, there were three groups at each center. The allocation sequence was known only to an independent statistical specialist. To randomize participants, a file containing the participant’s research identification number was emailed to the statistical specialist by the principal investigator or study coordinator at the participating center, and a file containing the participant’s assignment information was received via email from the statistical specialist. Cognitive outcome assessors were blinded to the assigned groups and were not involved in the intervention activities. The participants were advised not to discuss the intervention during the testing sessions.

The participants from the FMI and HMI groups received interventions of the five components previously described in detail [8, 10], including monitoring and management of metabolic and vascular risk factors, cognitive training and social activity, physical exercise [9], nutritional guidance, and motivational enhancement.

Participants in the FMI group visited a facility three times per week to participate in all intervention programs in the group or individual sessions. The contents of the cognitive training and physical exercise programs in the HMI group were identical to those in the FMI group. Those in the HMI arm participated in one group of cognitive training sessions (each lasting 50 min) and one home-based cognitive training session (each lasting 30–40 min) per week and one group of exercise sessions (each lasting 60 min) and two home-based exercise sessions (each lasting 60 min) per week during the first 2 months of the trial. For the remainder of the 6-month study, participants in the HMI arm attended one group cognitive training session and one group exercise session every two weeks. For the weeks that included group sessions, the participants performed one cognitive training session and two exercise sessions alone at home each week. For weeks that did not include group sessions, participants performed two cognitive training sessions and three exercise sessions alone at home each week. The control group received regular health advice according to the established guidelines.

### Cognitive Outcomes and Serum Brain-Derived Neurotrophic Factor

The total scale index score of the Repeatable Battery for the Assessment of Neuropsychological Status (RBANS) was assessed at baseline and within 4 weeks after the last intervention in all participants. The RBANS consists of tests A, B, C, and D, with an identical degree of difficulty. Each test included 12 subtests and evaluated the following five cognitive domains: attention, language, visuospatial/constructional abilities, immediate memory, and delayed memory [31]. The participants performed tests A and D of the RBANS at baseline and post-intervention, respectively. A standard score based on same-aged peers for each of the five cognitive domains was provided. These indices were combined to compute the total scale scores of cognitive functioning. Higher scores indicated better cognitive functioning.

Changes in serum BDNF levels after multidomain intervention were investigated. Fasting blood samples were collected at around 9 a.m. in serum separator tubes (SSTs) within 4 weeks before the intervention and within 4 weeks after the last intervention. The SSTs were maintained at room temperature for 30 min and then centrifuged for 10 min at 3000 rpm. In 0.5 mL aliquots, serum samples were stored in cryovial tubes at ≤ −70 °C until analysis. Serum BDNF levels were measured using a quantitative sandwich enzyme-linked immunosorbent assay (ELISA) using DBD00 (R&D Systems, Inc., Minneapolis, MN, USA), according to the manufacturer’s instructions.

### MRI Acquisition and Processing

Brain MRI scans at baseline and follow-up were acquired at three clinical sites: Ajou University Hospital, Ewha Woman’s University Medical Center, and Inha University Hospital. The following MR systems and 3D T1 imaging parameters were used: 3 T Achieva, Philips (sagittal slice thickness, 1.2 mm; no gap; repetition time (TR), 6.9 ms; echo time (TE), 3.2 ms; flip angle, 9°; voxel size 1.2 × 1.0 × 1.0 mm^3^ and a matrix size of 256 × 256 pixels) at the Ajou University Hospital, 3 T Achieva, Philips (sagittal slice thickness, 1.2 mm; no gap; TR, 7.9 ms; TE, 3.7 ms; flip angle, 9°; voxel size 1.2 × 1.0 × 1.0 mm^3^ and a matrix size of 256 × 256 pixels) at the Ewha Woman’s University Medical Center, and 3 T Signa Architect, GE, (sagittal slice thickness, 1.2 mm; no gap; TR, 7.4 ms; TE, 2.7 ms; flip angle, 11°; voxel size 1.2 × 1.0 × 1.0 mm^3^ and a matrix size of 256 × 256 pixels) at the Inha University Hospital. The same imaging parameters and MRI scanners were used for both the baseline and 24-week scans at each site. Regular phantom scans were performed within each site, although the phantom was not shared among three centers. Before quantitative analysis, an experienced neurologist visually inspected the T1-weighted images at each site. Images were excluded if there were brain lesions potentially affecting volumetry and/or scanning issues, such as no full-brain coverage, artifacts, intensity inhomogeneity, and adequate gray/white matter contrast.

We used the CIVET v2.1 pipeline (https://wiki.bic.mni.mcgill.ca/ServicesSoftware/CIVET) developed by the Montreal Neurological Institute (MNI) to measure cortical thickness, as described in detail elsewhere [32, 33]. The native T1-weighted images were corrected for intensity inhomogeneity and spatially normalized to the MNI-152 symmetric template [34, 35]. The corrected and registered images were segmented into gray matter, white matter, cerebrospinal fluid, and background using a 3D stereotaxic brain mask and the Intensity-Normalized Stereotaxic Environment for Classification of Tissues (INSECT) algorithm.

Further, the hemispherical gray matter and white matter surfaces, consisting of 40,962 vertices, were extracted from each MR volume using the constrained Laplacian-based automated segmentation with Proximities (CLASP) algorithm [36, 37]. Finally, the cortical thickness was measured in native space by calculating the Euclidean distance between the corresponding vertices of the inner and outer surfaces [37–39]. The global cortical thickness was calculated as the average cortical thickness of all 81,924 vertices. For the region-of-interest (ROI)-based analysis, two types of the atlas were used: the cortex was divided into eight regions using a lobe atlas (https://www.bic.mni.mcgill.ca/ServicesSoftware/VisualGuides) and 76 regions using the automated anatomical labeling (AAL) atlas. The average values of the thickness of the entire vertex in each region were used for our analysis.

### Statistical Analyses

To verify if there were differences between groups at baseline, we performed a *t*-test for independent samples, and the chi-square test for comparison of categorical variables (FMI vs. control; HMI vs. control; FMI vs. HMI). Analysis of covariance (ANCOVA), with a baseline score as a covariate, was used to compare changes from baseline to the study endpoint in the RBANS and serum BDNF levels between each intervention group and the control group. The groups were considered similar when *p* > 0.05. In addition, correlation analyses between changes of the RBANS and serum BDNF levels were done by the Pearson analyses.

Changes in total cortical gray matter volume, mean cortical thickness at each ROI, and global mean cortical thickness were compared between each group pair (FMI vs. control; HMI vs. control; FMI vs. HMI) using ANCOVA with changes as dependent variables and age, sex, and education as covariates. In addition, changes of cortical thickness in AD signature regions calculated as the average of cortical thickness in entorhinal, inferior temporal, middle temporal, and fusiform regions as previously described [40] was also compared. Finally, correlation analyses between changes of the RBANS or the serum BDNF levels and changes in total cortical gray matter volume, mean cortical thickness at each ROI, and global mean cortical thickness were done by the Pearson analyses, respectively. ComBat site harmonization was also applied to combine and harmonize cortical thickness values across the scanners [41–43]. Specifically, the statistical analyses were conducted using statsmodels.api.OLS package (https://www.statsmodels.org/dev/generated/statsmodels.regression.linear_model.OLS.html). We used SurfStat (http://www.math.mcgill.ca/keith/surfstat/) for cortical thickness visualization and analysis with a false discovery rate threshold of *p* < 0.05 to control for multiple comparisons.

## Results

The population undergoing MRI was younger, more educated, and had a higher baseline RBANS total scale index than the population not undergoing MRI in SUPERBRAIN (Supplementary Table 1). In contrast, their adherence to the intervention or distribution of sex, APOE ε4 carriers, or distribution of groups was not different. The intervention and control groups in the SUPERBRAIN exploratory MRI sub-study were not significantly different in terms of demographic, clinical, cognitive, and MRI characteristics at the baseline (Table 1). The adherence rates in the FMI and HMI groups were 96.0% and 97.0%, respectively. The total cortical gray matter volume, mean cortical thickness at each ROI, global, and AD signature mean cortical thickness at the baseline were not different among groups (Tables 1, 2, and 3).

**Table 1:** Baseline clinical characteristics and their changes after 24 weeks in the intervention and control groups

	**FMI (*n* = 20)**	**HMI (*n* = 19)**	**Control (*n* = 16)**	***p*, FMI *vs.* control**	***p*, HMI *vs.* control**	***p*, FMI *vs.* HMI**
**Baseline**						
Age, y	68.6 ± 4.9	68.0 ± 4.4	67.1 ± 4.4	0.367	0.607	0.687
Education, y	11.3 ± 4.2	11.8 ± 4.4	9.8 ± 4.3	0.294	0.161	0.696
Female, *n* (%)	14 (70.0)	12 (63.2)	14 (87.5)	0.257	0.135	0.676
APOE *ε*4 carriers, *n* (%)	3 (15.0)	2 (10.5)	3 (18.8)	1.000	0.642	0.352
Total intracranial volume, mL	1356.7 ± 1353.9	1345.7 ± 1299.8	1314.0 ± 1468.4	0.358	0.499	0.804
RBANS indexes						
Total	109.6 ± 19.1	110.4 ± 14.1	105.5 ± 18.8	0.488	0.411	0.884
Attention	112.3 ± 16.5	112.9 ± 14.5	104.8 ± 18.1	0.179	0.152	0.910
Immediate recall	106.1 ± 15.7	104.1 ± 16.4	101.1 ± 15.2	0.356	0.584	0.697
Delayed recall	99.7 ± 18.3	98.2 ± 11.8	99.9 ± 17.4	0.965	0.761	0.782
Visuospatial/Construction	99.1 ± 11.1	101.5 ± 14.2	95.6 ± 13.4	0.436	0.186	0.553
Language	108.7 ± 15.2	111.8 ± 10.3	111.2 ± 15.1	0.582	0.899	0.478
BDNF, ng/mL	29.9 ± 11.4	33.5 ± 15.6	40.5 ± 22.1	0.062	0.215	0.508
**Changes over 24-weeks**						
RBANS indexes						
Total	7.1 ± 6.4	5.2 ± 9.7	−2.3 ± 9.8	0.002	0.013	0.491
Attention	1.5 ± 8.2	−0.5 ± 9.0	−2.7 ± 9.1	0.156	0.460	0.475
Immediate memory	6.2 ± 9.4	7.3 ± 14.1	3.3 ± 10.4	0.461	0.319	0.774
Delayed memory	10.6 ± 10.3	11.0 ± 9.3	2.9 ± 7.9	0.017	0.014	0.908
Visuospatial/Construction	3.0 ± 9.3	−0.9 ± 16.4	−8.4 ± 13.4	0.013	0.103	0.359
Language	2.1 ± 12.1	0.1 ± 11.5	−3.1 ± 11.1	0.187	0.411	0.605
BDNF, ng/mL	14.7 ± 24.6	4.2 ± 2.2	−3.7 ± 2.6	0.029	0.338	0.188

*FMI* facility-based multidomain intervention, *HMI* home-based multidomain intervention, *RBANS* Repeatable Battery for the Assessment of Neuropsychological Status

**Fig. 2:**
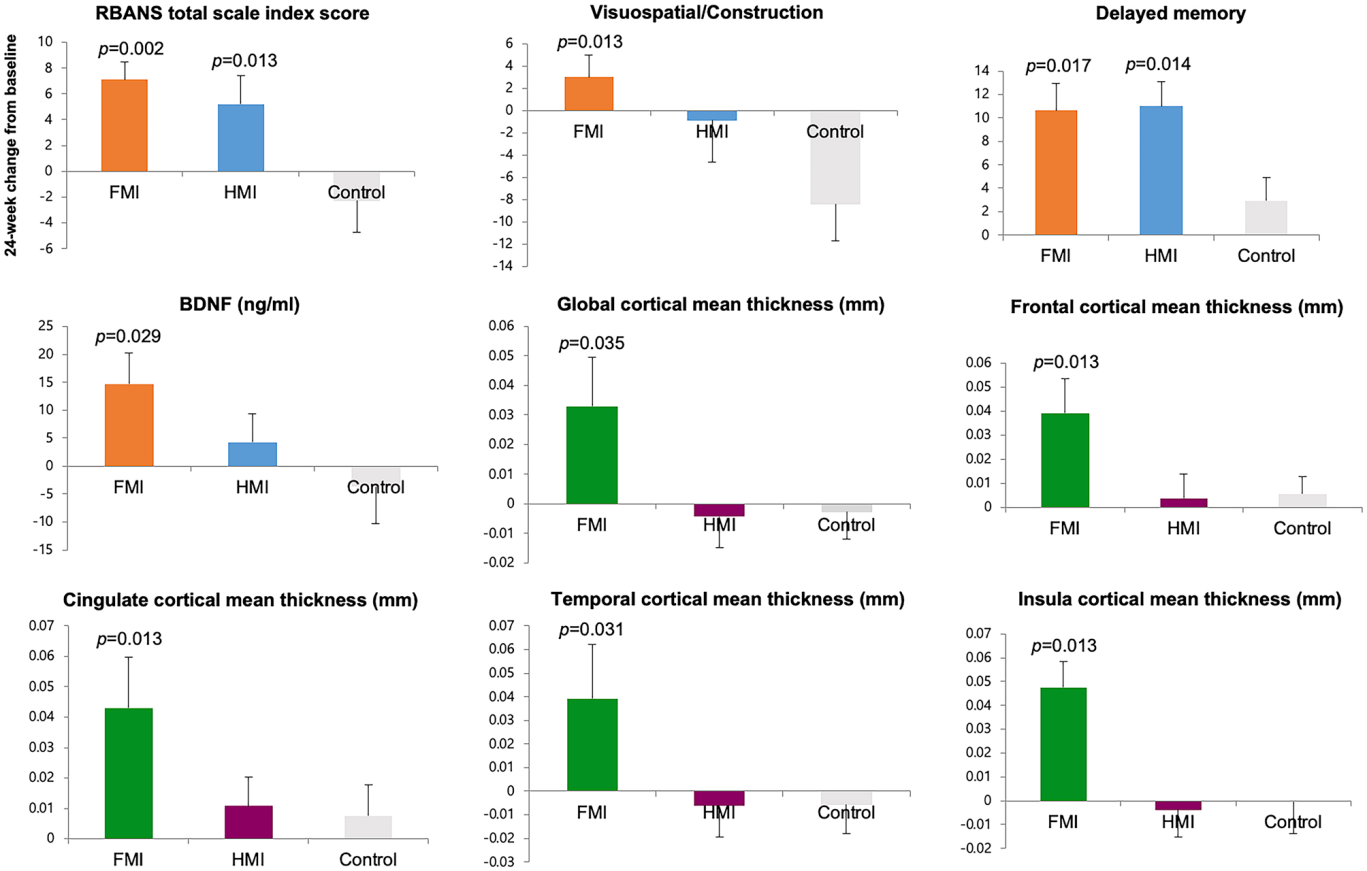
Mean changes from baseline at study end in the RBANS index score, a blood biomarker and cortical thickness. The bars and lines represent the mean and standard error of the mean. The *p* values represent the results of comparison between the intervention and control groups using analysis of covariance. FMI facility-based multidomain intervention, HMI home-based multidomain intervention

Compared with the control group, the total scale index score of the RBANS was significantly improved in both the FMI (*p* = 0.002) and HMI groups (*p* = 0.013) at the end of the study (Table 1; Fig. 2). In the FMI group, compared with the control group, delayed memory (*p* = 0.017) and visuospatial/construction (*p* = 0.013) revealed significantly greater improvement among the components of the RBANS. In the HMI group, only delayed memory showed significantly greater improvement than in the control group (*p* = 0.014). Serum BDNF levels were significantly higher in the FMI group than in the control group (*p* = 0.029; Table 1; Fig. 2). There was no significant difference in the change of the total scale index and five cognitive domains scores of the RBANS as well as serum BDNF levels between the FMI and HMI groups. The correlation analyses did not show any significant correlation between changes of the RBANS and serum BDNF levels.

**Fig. 3:**
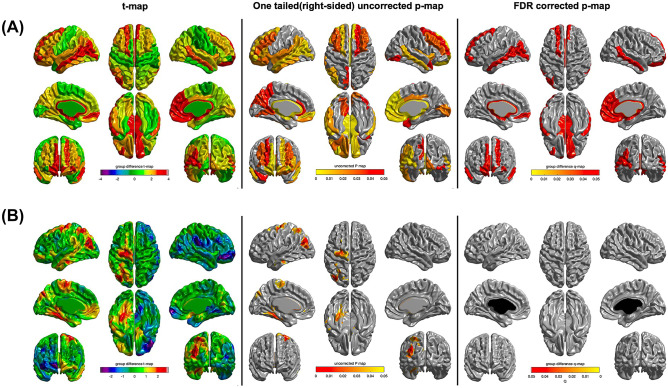
Effects of intervention on cortical thickness among AAL regions (analysis with a false discovery rate threshold of *p* < 0.05 to control for multiple comparisons) in each intervention group in comparison to the control group. The FMI group (**A**) yielded significantly more beneficial effects on the cortical thickness of the dorsolateral part of the left superior frontal gyrus, orbital part of the left middle frontal gyrus, medial orbital part of left superior frontal gyrus, left middle occipital gyrus, left middle temporal gyrus, orbital part of right superior frontal gyrus, the medial part of right superior frontal gyrus, medial orbital part of right superior frontal gyrus, right olfactory cortex, right gyrus rectus, right anterior cingulate and paracingulate gyri, and right middle temporal gyrus. However, the changes in the mean regional cortical thickness were not significantly different between the HMI and control groups (**B**). FMI facility-based multidomain intervention, HMI home-based multidomain intervention

The global cortical thickness as well as regional cortical thickness in the occipital, frontal, cingulate, temporal, and insular regions among the eight ROIs using the lobe atlas increased more significantly only in the FMI group than in the control group, whereas change of the total cortical gray matter volume were not different between the groups (Fig. 2; Table 2). However, the changes in the mean global cortical thickness and cortical thickness among the eight ROIs using the lobe atlas were not significantly different between the HMI and control groups. Among the AAL regions, compared with the control group, the FMI group yielded significantly more beneficial effects on the cortical thickness of the dorsolateral part of the left superior frontal gyrus, orbital part of left middle frontal gyrus, medial orbital part of left superior frontal gyrus, left middle occipital gyrus, left middle temporal gyrus, orbital part of right superior frontal gyrus, medial part of right superior frontal gyrus, medial orbital part of right superior frontal gyrus, right olfactory cortex, right gyrus rectus, right anterior cingulate and paracingulate gyri, and right middle temporal gyrus (Table 3; Fig. 3). Finally, mean cortical thickness in AD signature regions tended to be more increased after the intervention in the FMI group than the control (Table 2; *p* = 0.054).

**Table 2:** Effects of the intervention on the mean cortical thickness of the global or lobar areas

	Baseline, mm			Changes (from baselines to study end), mm			*p-*value*		
Cortical areas	FMI (*n* = 20)	HMI (*n* = 19)	Control (*n* = 16)	FMI (*n* = 20)	HMI (*n* = 19)	Control (*n* = 16)	FMI vs. Control	HMI vs. Control	FMI vs.HMI
**Global mean**	3.095 (0.090)	3.108 (0.080)	3.126 (0.110)	0.033 (0.070)	−0.004 (0.050)	−0.003 (0.040)	0.013	0.732	0.050
**AD signature region**	3.529 (0.253)	3.529 (0.246)	3.578 (0.272)	0.031 (0.140)	−0.001 (0.101)	−0.000 (0.117)	0.054	0.726	0.192
8 ROI using lobe atlas*									
Parietal	2.885 (0.117)	2.93 (0.104)	2.949 (0.119)	0.026 (0.077)	−0.001 (0.065)	0.002 (0.047)	0.0723	0.4548	0.158
Occipital	2.951 (0.151)	2.925 (0.142)	2.921 (0.157)	0.009 (0.124)	−0.044 (0.081)	−0.04 (0.063)	0.0418	0.4548	0.120
Frontal	3.095 (0.093)	3.106 (0.108)	3.129 (0.108)	0.039 (0.063)	0.004 (0.042)	0.006 (0.028)	0.0095	0.4548	0.048
Isthmus	3.325 (0.19)	3.315 (0.117)	3.358 (0.207)	0.026 (0.107)	0.001 (0.069)	−0.006 (0.083)	0.0583	0.4548	0.199
Parahippocampal	3.32 (0.134)	3.339 (0.078)	3.402 (0.126)	0.035 (0.088)	0.01 (0.043)	0.007 (0.06)	0.0723	0.4548	0.161
Cingulate	3.115 (0.139)	3.098 (0.084)	3.144 (0.133)	0.043 (0.073)	0.011 (0.04)	0.008 (0.039)	0.0085	0.4548	0.048
Temporal	3.354 (0.097)	3.369 (0.094)	3.391 (0.121)	0.039 (0.101)	−0.006 (0.055)	−0.006 (0.048)	0.0145	0.4548	0.081
Insula	3.635 (0.108)	3.638 (0.139)	3.682 (0.174)	0.048 (0.047)	−0.004 (0.048)	−0.0 (0.052)	0.0085	0.4548	0.002

Data are presented as the mean (standard deviation)

*AD* Alzheimer’s disease, *FMI* facility-based multidomain intervention, *HMI* home-based multidomain intervention

^*^Analysis with a false discovery rate threshold of *p* < 0.05 to control for multiple comparisons

**Table 3:** Areas showing beneficial effects of the intervention on mean cortical thickness among the AAL regions

	Baseline, mm			Changes (from baselines to study end), mm			*p*-value*		
	FMI (*n* = 20)	HMI (*n* = 19)	Control (*n* = 16)	FMI (*n* = 20)	HMI (*n* = 19)	Control (*n* = 16)	FMI vs Control	HMI vs Control	FMI vs HMI
Dorsolateral part of left superior frontal gyrus	3.049 (0.096)	3.057 (0.141)	3.082 (0.128)	0.034 (0.072)	0.001 (0.073)	-0.023 (0.04)	0.0443	0.8444	0.1737
Orbital part of left middle frontal gyrus	3.076 (0.119)	3.093 (0.102)	3.154 (0.171)	0.05 (0.061)	0.003 (0.058)	-0.025 (0.066)	0.0429	0.8444	0.1092
Medial orbital part of left superior fontal gyrus	3.186 (0.109)	3.141 (0.095)	3.227 (0.154)	0.077 (0.166)	0.02 (0.057)	-0.012 (0.094)	0.0429	0.8444	0.0671
Left middle occipital gyrus	3.057 (0.173)	3.027 (0.178)	3.08 (0.186)	0.023 (0.167)	-0.036 (0.109)	-0.076 (0.085)	0.0429	0.8444	0.1854
Left middle temporal gyrus	3.268 (0.112)	3.328 (0.107)	3.351 (0.173)	0.033 (0.119)	-0.026 (0.085)	-0.058 (0.103)	0.0429	0.8444	0.0452
Orbital part of right superior frontal gyrus	3.142 (0.154)	3.165 (0.111)	3.191 (0.127)	0.077 (0.131)	0.014 (0.068)	0.028 (0.081)	0.0429	0.8901	0.1718
Medial part of right superior frontal gyrus	3.245 (0.115)	3.258 (0.153)	3.284 (0.114)	0.068 (0.086)	-0.004 (0.051)	-0.003 (0.059)	0.0429	0.8571	0.0671
Medial orbital part of right superior frontal gyrus	3.191 (0.145)	3.233 (0.12)	3.257 (0.13)	0.073 (0.129)	-0.01 (0.065)	0.023 (0.08)	0.0429	0.9332	0.1727
Right olfactory cortex	2.934 (0.196)	2.938 (0.113)	2.979 (0.156)	0.108 (0.157)	0.053 (0.117)	0.022 (0.108)	0.0429	0.8444	0.0671
Right gyrus rectus	3.135 (0.201)	3.158 (0.114)	3.193 (0.129)	0.109 (0.196)	0.037 (0.126)	0.034 (0.117)	0.0429	0.8444	0.2278
Right anterior cingulate and paracingulate gyri	3.101 (0.215)	3.077 (0.152)	3.125 (0.143)	0.074 (0.091)	0.011 (0.054)	0.011 (0.083)	0.0429	0.8501	0.1276
Right middle temporal gyrus	3.31 (0.101)	3.319 (0.129)	3.309 (0.162)	0.08 (0.094)	0.008 (0.074)	0.022 (0.068)	0.0491	0.8901	0.0671

Data are presented as the mean (standard deviation)

*FMI* facility-based multidomain intervention, *HMI* home-based multidomain intervention, *L* left, *R* right

^*^Analysis with a false discovery rate threshold of *p* < 0.05 to control for multiple comparisons

**Fig. 4:**
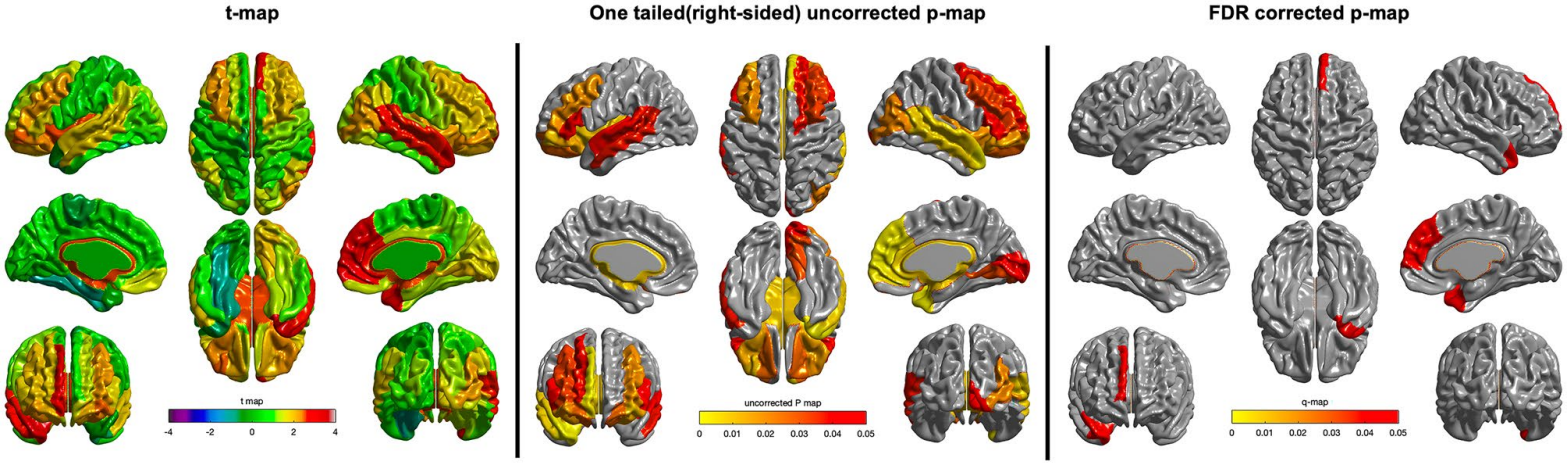
Effects of intervention on cortical thickness among AAL regions (analysis with a false discovery rate threshold of *p* < 0.05 to control for multiple comparisons) in the FMI group in comparison to the HMI group. Compared with the HMI, the FMI group yielded significantly more beneficial effects on the cortical thickness of right medial superior frontal gyrus (*p* = 0.045), right temporal pole of superior temporal gyrus (p = 0.045) and middle temporal gyrus (*p* = 0.045) among the AAL regions. FMI facility-based multidomain intervention, HMI home-based multidomain intervention

In comparison between the FMI and HMI groups, mean global cortical thickness tended to be more increased after the intervention in the FMI group than the HMI group (Table 2; *p* = 0.050). In addition, compared with the HMI, the FMI group yielded significantly more beneficial effects on the cortical thickness of frontal, cingulate, and insular regions among the eight ROIs using the lobe atlas (Table 2) and right medial superior frontal gyrus (*p* = 0.045), right temporal pole of superior temporal gyrus (*p* = 0.045), and middle temporal gyrus (*p* = 0.045) among the AAL regions (Table 3; Fig. 4). Finally, changes of the mean cortical thickness in AD signature regions were not different between two intervention groups (Table 2; *p* = 0.192).

The correlation analyses did not show any significant correlation between changes of the RBANS or the serum BDNF levels and changes in total cortical gray matter volume, mean cortical thickness at each ROI, and global mean cortical thickness.

## Discussion

To the best of our knowledge, this study is the first to show that multidomain lifestyle intervention for dementia prevention could be beneficial for global and regional cortical thickness. Compared with the control group, the FMI group showed significantly increased cortical thickness after the 6-month intervention.

Increased global and regional cortical thickness in the FMI group suggests that FMI programs, such as those used in the current study, may counteract age-associated structural changes in the elderly. In particular, a previous study showed that the lateral and medial prefrontal cortices, as well as the superior and middle temporal gyri, were consistently affected during the aging process [44]. The precise neurobiological mechanisms that are responsible for the changes in cortical thickness in older adults after the FMI program are not known. However, previous studies have suggested several explanations, such as changes in the size of neurons or glial cells, as well as the genesis of neurons or glial cells or changes in vascularization [45–48]. This is thought to be mediated by increased serum levels of BDNF, insulin-like growth factor type-1, and vascular endothelial growth factors, often noted after physical exercise [49]. In our study, compared with the control group, the FMI group showed an increase in the serum BDNF levels and cortical thickness, whereas the HMI group revealed no significant difference in the serum BDNF levels and cortical thickness. Because there was no significant correlation between the serum BDNF levels and changes in the mean cortical thickness, our study could not reveal that increased cortical thickness after the FMI program in the current study was related to increased serum BDNF levels. However, increased cortical thickness as well as serum BDNF after our FMI program showed that our FMI program could be neuroplastic. Further, the population undergoing MRI was younger, more educated, and had a higher baseline RBANS total scale index than the population not undergoing MRI in the SUPERBRAIN. In younger and/or high-performing populations, a more powerful intervention might be necessary to provoke plastic changes in the cortices. The FMI program involved more face-to-face intervention with interaction within group meetings, and therefore, this FMI could provide more stimuli for plastic changes in the brain than HMI as shown in comparison of the changes in the mean cortical thickness at each ROI between the FMI and HMI groups.

In the analyses of the change in regional cortical thickness, compared with the control group, the FMI group yielded significantly more beneficial effects on the cortical thickness of the occipital, frontal, cingulate, temporal, or insula among eight ROIs using the lobe atlas and the dorsolateral part of the left superior frontal gyrus, orbital part of the left middle frontal gyrus, medial orbital part of the left superior frontal gyrus, left middle occipital gyrus, left middle temporal gyrus, orbital part of the right superior frontal gyrus, the medial part of the right superior frontal gyrus, medial orbital part of the right superior frontal gyrus, right olfactory cortex, right gyrus rectus, right anterior cingulate and paracingulate gyri, and right middle temporal gyrus among the AAL regions. These areas are heteromodal association cortices responsible for integrating information from the unimodal association cortex and the paralimbic areas, which are important in facilitating learning [50, 51]. Compared with the rest of the cortex, these regions exhibit a distinct gene expression profile characterized by relative upregulation of gene sets implicated in ionotropic and metabotropic neurotransmission as well as activation of immune response and underlie a higher capacity for plastic changes in response to lifetime intellectual enrichment and a potential higher resilience to age-related pathologic brain changes [51]. The programs used in this study may require strong integration of information as well as active interactions between neuronal networks because these interventions target multiple cognitive domains, which require activation and processing by the heteromodal association cortices. In addition, these areas include regions which are known to be AD signature areas and our study showed that mean cortical thickness in AD signature regions tended to be more increased after the intervention in the FMI group than the control; therefore, increased cortical thickness in these areas might be beneficial in the prevention of cognitive decline in the AD.

In contrast to the cognitive benefits of multidomain lifestyle interventions, the effects of lifestyle interventions on cortical thickness or brain volumes have not yet been fully clarified. While the FINGER trial revealed significant cognitive benefits of a multidomain lifestyle intervention [4], the FINGER MRI exploratory sub-study did not show significant differences between the intervention and control groups in terms of changes in brain volume or regional cortical thicknesses after 2 years in at-risk elderly without substantial impairment [21]. It had been thought that the negative results on the cortical thickness in the FINGER MRI exploratory sub-study might be due to the characteristics of the study population. The participants were at-risk segments of the general elderly population (not patients in a clinical setting), and their cortical thickness was approximately decreased by 0.1 mm during 2 years in both the intervention and control groups. The structural brain changes in this at-risk population were not very pronounced over 2 years, and this interval may not have been sufficient to observe significant effects on structural brain changes. In our study, the intervention lasted for 6 months, during which the global cortical thickness in the FMI group increased by approximately 0.033 mm, whereas the control group showed a decrease in global cortical thickness by 0.003 mm. It is impossible to directly compare the results from two studies, the FINGER and SUPERBRAIN, due to the difference in the trial duration. Smaller (*N* < 160 participants) and/or shorter trials (up to 24 weeks) seemed more likely to report intervention benefits on overall cognition and some specific domains (e.g., spatial working memory, executive functioning) [52]. Therefore, the distinct beneficial impact of the 6-month FMI of the SUPERBRAIN on the cortical thickness could not guarantee longer-term effects of multidomain lifestyle interventions, especially as some older at-risk people may start to develop brain pathology. Therefore, further investigation is needed to determine whether the impact of multidomain lifestyle interventions with high adherence on brain volume or cortical thickness would simply disappear or be sustainable in the longer-term.

The main limitation of the SUPERBRAIN MRI exploratory sub-study is the small sample size together with the short trial because this study was a SUPERBRAIN MRI exploratory sub-study, and the results should be interpreted with caution. Future studies including cortical thickness as a primary outcome measure are necessary to confirm the impact of multidomain lifestyle intervention on cortical thickness in the longer-term. In addition, MRI scanners differed among sites; however, this was adjusted for in all analyses.

In conclusion, significant differences between the FMI and control groups in changes in cortical thickness in at-risk elderly individuals without substantial impairment in the SUPERBRAIN suggest that group preventive strategies at the facility may be beneficial for cognition through structural neuroplastic changes in the brain areas, which play a crucial role in facilitating learning. Future studies, including cortical thickness as a primary outcome measure, are necessary to confirm the impact of multidomain lifestyle intervention on cortical thickness.

## Supplementary Information

Below is the link to the electronic supplementary material.

Supplementary file1 (PPTX 41 kb)

Supplementary file2 (PDF 533 kb)

Supplementary file3 (PDF 534 kb)

Supplementary file4 (PDF 551 kb)

Supplementary file5 (PDF 543 kb)

Supplementary file6 (PDF 579 kb)

Supplementary file7 (PDF 508 kb)

Supplementary file8 (PDF 559 kb)

Supplementary file9 (PDF 542 kb)

Supplementary file10 (PDF 517 kb)

Supplementary file11 (PDF 570 kb)

Supplementary file12 (PDF 525 kb)

Supplementary file13 (PDF 570 kb)

Supplementary file14 (PDF 516 kb)

## Declarations

### Disclaimer

The funders had no role in the study design, data collection, analysis, interpretation of data, writing of the report, or decision to submit the manuscript for publication.
