# Supplementary material for: Impact of Multidomain Lifestyle Intervention on Cerebral Cortical Thickness and Serum Brain-Derived Neurotrophic Factor: the SUPERBRAIN Exploratory Sub-study
Source: Neurotherapeutics. 2022 Aug 1;19(5):1514–25. doi: 10.1007/s13311-022-01276-x (PMC9606175; doi:10.1007/s13311-022-01276-x)
Supplement: Supplementary file 1 — Supplementary file1 (PPTX 41 kb) [file 13311_2022_1276_MOESM1_ESM.pptx]

## Slide 1
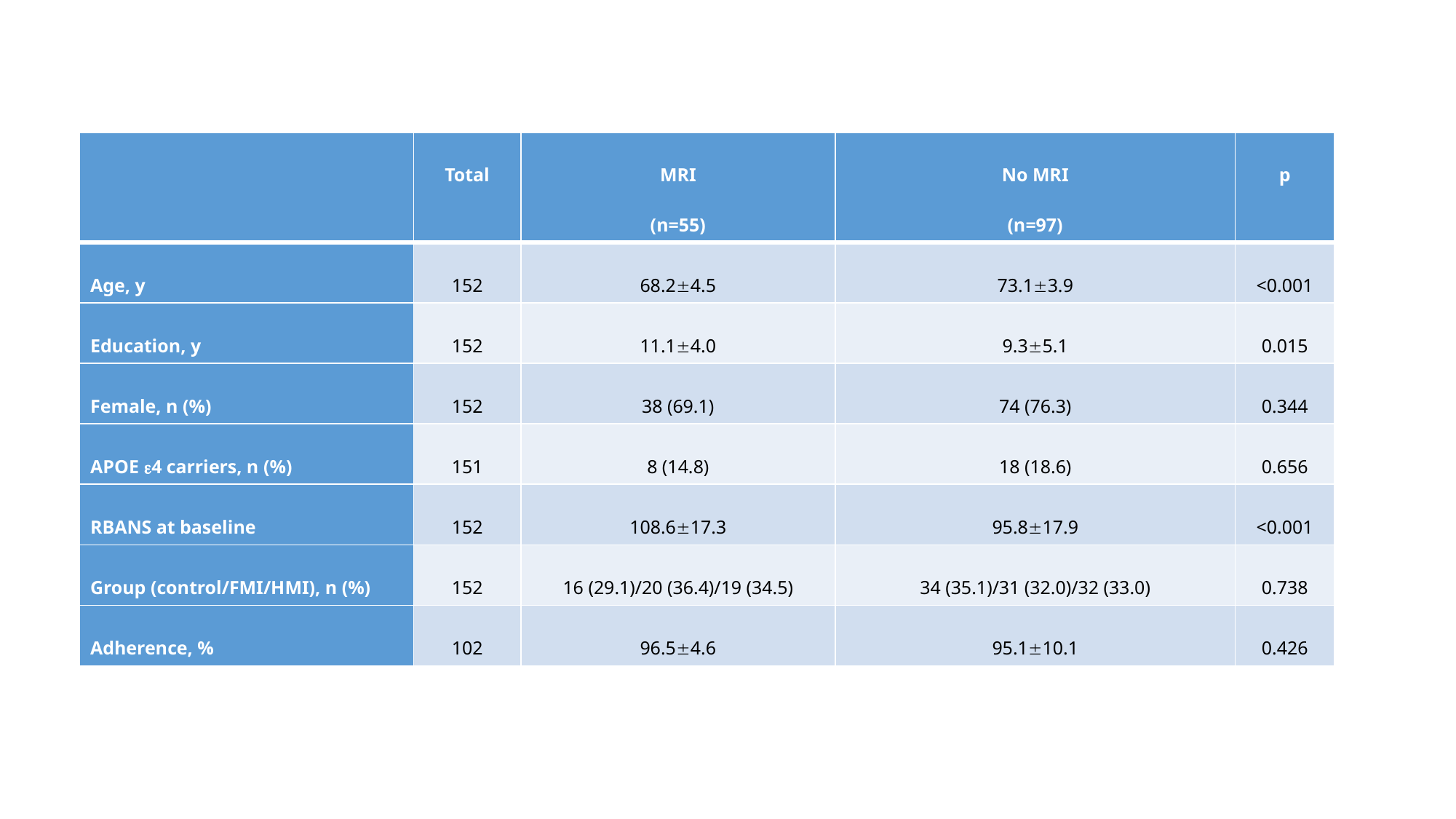

| | Total | MRI (n=55) | No MRI (n=97) | p |
| --- | --- | --- | --- | --- |
| Age, y | 152 | 68.2±4.5 | 73.1±3.9 | <0.001 |
| Education, y | 152 | 11.1±4.0 | 9.3±5.1 | 0.015 |
| Female, n (%) | 152 | 38 (69.1) | 74 (76.3) | 0.344 |
| APOE ε4 carriers, n (%) | 151 | 8 (14.8) | 18 (18.6) | 0.656 |
| RBANS at baseline | 152 | 108.6±17.3 | 95.8±17.9 | <0.001 |
| Group (control/FMI/HMI), n (%) | 152 | 16 (29.1)/20 (36.4)/19 (34.5) | 34 (35.1)/31 (32.0)/32 (33.0) | 0.738 |
| Adherence, % | 102 | 96.5±4.6 | 95.1±10.1 | 0.426 |
